# Resistant Potato Starch Supplementation Reduces Serum Free Fatty Acid Levels and Influences Bile Acid Metabolism

**DOI:** 10.3390/metabo14100536

**Published:** 2024-10-05

**Authors:** Jason R. Bush, Izuchukwu Iwuamadi, Jun Han, David J. Schibli, David R. Goodlett, Edward C. Deehan

**Affiliations:** 1 MSP Starch Products Inc., Carberry, MB R0K 0H0, Canada; 2 Department of Food Science and Technology, University of Nebraska, Lincoln, NE 68588, USA; iiwuamadi2@huskers.unl.edu (I.I.); edeehan2@unl.edu (E.C.D.); 3 Nebraska Food for Health Center, University of Nebraska, Lincoln, NE 68588, USA; 4 UVic-Genome British Columbia Proteomics Centre, University of Victoria, Victoria, BC V8Z 7X8, Canada; hanjun@proteincentre.com (J.H.); dschibli@proteincentre.com (D.J.S.); goodlett@uvic.ca (D.R.G.); 5 Division of Medical Sciences, University of Victoria, Victoria, BC V8Z 7X8, Canada; 6 Department of Biochemistry and Microbiology, University of Victoria, Victoria, BC V8Z 7X8, Canada

**Keywords:** prebiotic, resistant starch, resistant potato starch, free fatty acid, bile acid, ketone

## Abstract

**Background**: Resistant starches, such as high-amylose maize starch and resistant potato starch (RPS), have prebiotic effects that are linked to improved metabolism at >15 g/day, but the effects at lower doses have not been reported. **Methods**: We performed an exploratory post hoc analysis of free fatty acids (FFAs), bile acids (BAs), and ketone bodies in serum previously collected from a randomized, double-blind, placebo-controlled clinical trial evaluating the effects of one- and four-week consumption of 3.5 g/day RPS versus a placebo using two-way ANOVA adjusted by pFDR. Associations between week 4 changes in FFAs, BAs, and ketone bodies were assessed by Pearson’s correlations. **Results**: RPS consumption reduced total FFAs relative to the placebo, including multiple unsaturated FFAs and octanedioic acid, with reductions in taurine- and glycine-conjugated secondary BAs also detected (*q* < 0.05). No changes in ketone bodies were observed (*q* > 0.05). Changes in 7-ketodeoxycholic acid (*r* = −0.595) and glycolithocholic acid (*r* = −0.471) were inversely correlated with treatment-induced reductions in FFAs for RPS but not the placebo, suggesting the effects were from the prebiotic. Shifts in β-hydroxybutyrate were further correlated with FFA changes in both treatments (*q* < 0.05). **Conclusions**: These findings demonstrate that low doses of RPS positively influence fatty acid metabolism in humans, reducing circulating levels of FFA and conjugated BAs.

## 1. Introduction

Type 2 diabetes (T2D) is characterized by increasing insulin resistance and concomitant elevations in serum blood glucose, typically determined by measuring fasting blood glucose or hemoglobin A1C (HbA1c) [1,2,3]. However, T2D diagnosis (typically HbA1c ≥ 6.5) occurs late in the progression of metabolic dysfunction, where the initial phase is characterized by elevated levels of insulin to maintain normal glycemic levels, followed by mildly elevated blood glucose and HbA1c levels (HbA1c: 5.7–6.4) in prediabetes [1,2,3]. Throughout this process, insulin resistance, characterized by systemic impairments in insulin signaling in tissues such as the hepatic, muscle, and adipose tissues, is the primary driver of disease progression [1,2,3]. Insulin acts in a tissue-specific manner, suppressing gluconeogenesis in the liver while promoting glucose uptake, storage, and/or utilization by adipocytes and myocytes [4]. Insulin also influences free fatty acid (FFA; also known as non-esterified fatty acid) levels, which are released from adipocytes following triglyceride hydrolysis [5]. Elevated FFA levels can contribute to insulin resistance by driving hepatic glucose production. Higher insulin concentrations are also required to suppress FFA release from adipocytes than that required to stimulate glucose uptake by adipocytes [4]. High levels of FFAs are therefore considered an early risk factor for the development of T2D and related co-morbidities [5]. An effective combination of lifestyle modifications and dietary changes has historically been prescribed to those at risk of developing T2D, but the growing prevalence of both prediabetes and T2D suggests that novel early interventions are required to more effectively address the epidemic [6].

Resistant starches (RSs) hold promise as one such dietary intervention for the attenuation of insulin resistance and reduction in T2D risk [7,8]. Resistant starch refers to “the sum of starch and starch-degradation products that, on average, reach the human large intestine” [9]. Resistant starch can be categorized into the following five types: RS1, physically inaccessible starch; RS2, native starch granules; RS3, retrograded starch; RS4, chemically modified starch; RS5, starch complexed with lipids [10]. Resistant starch type 2 and RS3 represent important sources of dietary fiber because they have historically been a part of the human diet [11] and studies demonstrate that they influence the composition of the gut microbiota in healthy people [12,13,14,15,16,17]. Resistant starches have been shown to exert multifaceted influences on host physiology related to T2D risk and disease progression, including positive effects on the gut microbiota, secretion of incretins, cholesterol levels, and blood glucose levels [7,18]. Specifically, clinical trials have demonstrated that forms of RS2 promote metabolic benefits, including increases in glucagon-like peptide-1 (GLP-1) [13,19,20,21], decreases in circulating insulin and insulin resistance [22,23,24,25,26], attenuating fasting glucose levels [26,27,28,29], and weight loss [13,30]. However, prohibitively high ingredient doses (>40 g of RS-containing material/day) have impaired the formulation of dietary supplements or functional foods containing sufficient RS to promote metabolic improvements and the effects of lower doses compatible with these product formulations are not well understood.

We previously reported that daily RS2 supplementation with native resistant potato starch (RPS) at 3.5 g for 4 weeks enriched *Bifidobacterium* and *Akkermansia* [17], reduced constipation- and diarrhea-associated bowel movements [17], decreased serum histamine levels [31], and altered metabolites previously associated with gut barrier function [31] without detectible changes in dietary intake [32]. While the effects of RPS supplementation on surrogate measures of insulin resistance, such as glucose, insulin, and GLP-1, were not captured during the randomized, double-blind, placebo-controlled trial, targeted metabolomics analyses of serum were performed to quantify circulating levels of FFAs, bile acids (BAs), and ketone bodies. Here, we determined the effects of low-dose RPS supplementation on these mechanistic measures previously shown to influence glucose metabolism [5,33,34]. Findings from this analysis will inform the use of lower, formulation-friendly dosages in future clinical trials that aim to determine the effects of RS supplementation on insulin resistance and, thus, the risk of developing T2D.

## 2. Materials and Methods

### 2.1. Investigational Products

The resistant potato starch (RPS) used in this study was Solnul^®^ (MSP Starch Products Inc., Carberry, MB, Canada), which is manufactured via a proprietary processing method from the slurry co-product of potato product processors and contains >60% RS by weight on an “as is” basis (AOAC 2002.02). Therefore, the 3.5 g dose of RPS contained 2.1 g of RS. RPS is a type 2 RS with a native starch granular structure that resists digestion [8,35]. The placebo used was Amioca™ TF (Ingredion, Brampton, ON, Canada), a high-amylopectin corn starch that is fully digestible, contains no detectable levels of RS [12,26], and has been previously shown to not affect the gut microbiota [36]. Investigational products were packaged in identical single-serving packets and labeled according to randomization codes from Nutrasource Pharmaceutical and Nutraceutical Services (Guelph, ON, Canada) by staff not involved in study data collection to ensure blinding, as previously described [17].

### 2.2. Study Participants

The study participants have been previously described in detail [17]. In brief, healthy adults aged 18–69 years of age with a body mass index (BMI) of 18.0 to 34.9 kg/m^2^ were recruited and enrolled in the study. Those with a BMI ≥ 35 kg/m^2^ were excluded as their health, and any related metabolic changes, could influence the results of the study independent of the intervention. Those with a diagnosis of irritable bowel syndrome, dyspepsia, significant gastrointestinal disorders, or other major diseases were also excluded. Eligible participants agreed to not consume any vitamins, minerals, or dietary supplements 14 days prior to the randomization visit until the completion of the study. Participants were counseled to follow their habitual diet throughout the study period and no changes in dietary intake were observed, as previously reported [32].

### 2.3. Clinical Trial Design and Execution

The design and execution of this study have been described previously in detail [17]. In brief, the study took place between 30 October 2019 and 6 January 2020 in Guelph, ON, Canada, and we recruited participants from the general population in Guelph and the surrounding area. The study protocol was approved by Canadian Shield Ethics Review Board (tracking number 19-10-001; Burlington, ON, Canada) and registered at ClinicalTrials.gov (NCT05242913). Written informed consent was obtained from all study participants or their legally authorized representative prior to enrollment into the study following the Declaration of Helsinki and Council for International Organizations of Medical Sciences International Ethical Guidelines and ICH Good Clinical Practice guidelines [17].

The study was a randomized, double-blind, placebo-controlled, parallel-armed clinical trial that was designed to evaluate the effect of daily 3.5 g of RPS (containing 3.5 g of RPS and 3.5 g placebo, with 7 g total carbohydrates), 7 g of RPS, and 7 g of placebo for 4 weeks on fecal bacteria composition and bowel movement characteristics. Forty-eight participants completed the study protocol for the 3.5 g RPS (*n* = 24) and placebo (*n* = 24) arms. Non-fasting serum samples were collected at baseline and 4-weeks of supplementation and analyzed by targeted metabolomics to quantify circulating levels of FFAs, BAs, and ketone bodies. Due to commercial interest in the effects of low RPS doses, only the impact of 3.5 g RPS supplementation on mechanistic measures of fatty acid metabolism was assessed relative to the placebo.

### 2.4. FFA and Ketone Body Analysis

Quantitation of FFAs and ketone bodies was performed using the chemical derivatization liquid chromatography–multiple–reaction monitoring mass spectrometry (LC-MRM/MS) approach described previously [37,38], with necessary modifications and adaptations for FFAs and ketone bodies measured in this study. Stock solutions of FFAs and ketone bodies were prepared individually or in mixture with their standard substances in methanol or isopropanol. A mixed standard-substance solution was then prepared from the stock solutions and subsequently diluted step by step with methanol to obtain 9 calibration solutions. Next, 20 µL of each serum sample was aliquoted into a 1.5 mL safe-lock Eppendorf tube. To each 20 μL serum aliquot and to 20 µL of each calibration solution, 20 µL of an internal standard (IS) solution containing 10 isotope-labeled carboxylic acids of the tricarboxylic acid cycle [37] and 14 isotope-labeled, short to very long straight-chain fatty acids, which was prepared in methanol, 60 µL of 100 mM 3-nitrophenylhydrazine solution in 70% methanol and 60 µL of 100 mM 1-ethyl-3-(3-dimethylaminopropyl) carbodiimide-4% pyridine solution in methanol were added in turn. The mixtures were incubated at 50 °C for 60 min. After cooling down on ice for 1 min and centrifugation at 21,000× *g* for 10 min, the clear supernatants of sample solutions were transferred to LC injection microbials. Then, 3 µL aliquots of all the resultant samples and calibration solutions were used to run LC-MRM/MS on an Agilent 1290 ultrahigh-performance liquid chromatograph connected to an Agilent 6495B triple-quadrupole mass spectrometer (Agilent Technologies, Santa Clara, CA, USA), which was operated in the MRM mode and with negative-ion detection. An XBridge C8 column (2.1 × 50 mm, 2.5 µm; Waters Corp., Milford, MA, USA) and a mobile phase composed of 0.005% formic acid in water and 0.005% formic acid in acetonitrile-isopropanol (1:1) were used for binary-solvent gradient elution under optimized conditions for chromatographic separation and MRM/MS detection. Raw data files were acquired and processed using the Agilent MassHunter software suite (Agilent Technologies). Linear-regression calibration curves of individual FFAs and ketone bodies were constructed with the data acquired from their calibration solutions in an appropriate concentration range for each fatty acid. Concentrations of FFAs and ketone bodies in serum were calculated by interpolating the calibration curves with the analyte-to-IS peak area ratios measured from the sample solutions. For those analytes without their isotope-labeled analogues available or used in the assay, palmitic-d5 acid was used as a common IS.

Total saturated FFAs were determined as the sum of FA(7:0), FA(8:0), FA(9:0), FA(10:0), FA(11:0), FA(12:0), FA(13:0), FA(14:0), FA(15:0), FA(16:0), FA(17:0), FA(18:0), FA(19:0), FA(20:0), FA(21:0), FA(22:0), FA(23:0), FA(24:0), FA(25:0), and FA(26:0). Total unsaturated FFAs were determined as the sum of FA(8:1), FA(10:1), FA(12:1), FA(14:1), FA(16:1), FA(18:1), FA(18:2), FA(18:3), FA(18:4), FA(20:1), FA(20:2), FA(20:3), FA(20:4), FA(20:5), FA(22:1), FA(22:4), FA(22:5), FA(22:6), and FA(24:1). Total FFA levels were determined as the sum of all 39 individual FFAs noted above, plus FA(8:0-dicarboxyl), FA(10:0-dicarboxyl), FA(10:0-hydroxy), FA(14:0-hydroxy), and FA(16:0-hydroxy).

### 2.5. Bile Acid Analysis

Quantitation of BAs was carried out using the LC-MRM/MS method described previously [39], with necessary modifications for the sample preparation procedure and with the use of commercially acquired authentic compounds of 78 BAs as the standard substances. In brief, an IS solution of 16 deuterated BAs was prepared in a solvent of acetonitrile–methanol (2:1). A standard-substance solution of all the BAs, at 10 µM for each, was then prepared in a mixture of the IS solution–water (2:3). The standard solution was serially diluted with the same mixture in a ratio of 1 to 4 (*v*/*v*) to obtain 9 calibration solutions. For sample preparation, serum samples were thawed on ice and vortexed. Then, 20 µL of each was aliquoted into a 1.5 mL safe-lock Eppendorf tube. Next, 80 µL of the IS solution was added. The mixtures were vortexed for 15 s at 3000 rpm and then ultra-sonicated in an ice-water bath for 3 min, followed by centrifugal clarification at 21,000× *g* for 10 min. Following this, 80 µL of the clear supernatant of each sample was subsequently mixed with 40 µL of water in an LC injection micro-vial. After 3 s vortex mixing and 2 min centrifugation, 15 µL aliquots of the resultant sample and calibration solutions were used to run LC-MRM/MS on an Agilent 1290 ultrahigh-performance liquid chromatograph connected to a 4000 QTRAP mass spectrometer (AB Sciex LLC, Framingham, MA, USA), which was operated in the MRM mode and with negative-ion detection. A Waters BEH C18 column (2.1 × 150 mm, 1.7 µm; Waters Corp., Milford, MA, USA) and a mobile phase composed of 0.01% formic acid in water and 0.01% formic acid in acetonitrile were used for binary solvent gradient elution, according to the procedure and operating parameters described in the publication [39]. The raw data files were acquired using the Sciex Analyst software and processed using the Sciex MultiQuant 1.2 software (AB Sciex LLC, Framingham, MA, USA). Linear-regression calibration curves of individual BAs were constructed with the data acquired from the calibration solutions in an appropriate concentration range for each compound. Concentrations of BAs detected in serum were calculated by interpolating the calibration curves with the analyte-to-IS peak area ratios measured from the sample solutions. For those BAs without their deuterated forms as internal standard, glycodeoxycholic-d4 acid was used as a common IS.

Total glycine-conjugated BAs were determined as the sum of glycocholic acid, glycochenodeoxycholic acid, glycodeoxycholic acid, glycolithocholic acid, glycoursodeoxycholic acid, glycoallocholic acid, glyco-α-muricholic acid, glyco-β-muricholic acid, glycohyocholic acid, and glyco-ω-muricholic acid. Total taurine-conjugated BAs were determined as the sum of taurocholic acid, taurochenodeoxycholic acid, taurodeoxycholic acid, taurolithocholic acid, tauroursodeoxycholic acid, tauroallocholic acid, tauro-α-muricholic acid, taurohyocholic acid, and tauro-ω-muricholic acid. Total deconjugated BAs were determined as the sum of cholic acid, chenodeoxycholic acid, deoxycholic acid, lithocholic acid, ursodeoxycholic acid, allocholic acid, β-muricholic acid, hyocholic acid, ω-muricholic acid, isolithocholic acid, alloisolithocholic acid, 7-ketolithocholic acid, dioxolithocholic acid, dehydrolithocholic acid, 7-ketodeoxycholic acid, 12-ketochenodeoxycholic acid, 3-oxocholic acid, and ursocholic acid. Total BAs were determined as the sum of all individual BAs measured.

### 2.6. Statistical Analysis

Baseline, week 1, and week 4 levels of select FFAs, BAs, and ketone bodies were each compared between the placebo and RPS using two-way ANOVA to determine the overall effect of treatment and time. Changes in metabolite levels were further calculated by subtracting the baseline level from week 1 or week 4 levels. Mean changes in metabolite levels were then compared between the placebo and RPS using two-way ANOVA to assess the overall effect of treatment. Pearson correlation coefficients (*r*) were generated to assess associations between changes in select metabolites from baseline to week 4. To account for multiple comparisons, positive false discovery rate (pFDR) adjusted *q* values were generated using Storey’s method [40] at λ = 0.5 for the overall effect of treatment on metabolite changes as determined by two-way ANOVA and for Pearson correlations. Fisher’s exact test was used to compare the number of significant correlations between FFAs and ketone bodies, FFAs and BAs, and ketone bodies and BAs between groups. All statistical analyses were performed using Excel (Version 2409; Microsoft Excel, Redmond, WA, USA) except for Fisher’s exact tests (GraphPad Quick Calcs, Version 2024, GraphPad Software, Boston, MA, USA) and the Pearson correlations, which were performed using RStudio v4.3.2 (RStudio Team, Boston, MA, USA) with heatmaps of the correlation matrix generated using the ggplot2 package [41]. Statistical significance was considered at *p* < 0.05 or *q* < 0.05 when multiple comparisons were performed.

## 3. Results

### 3.1. RPS Decreases Circulating Free Fatty Acid Levels

**Figure 1:**
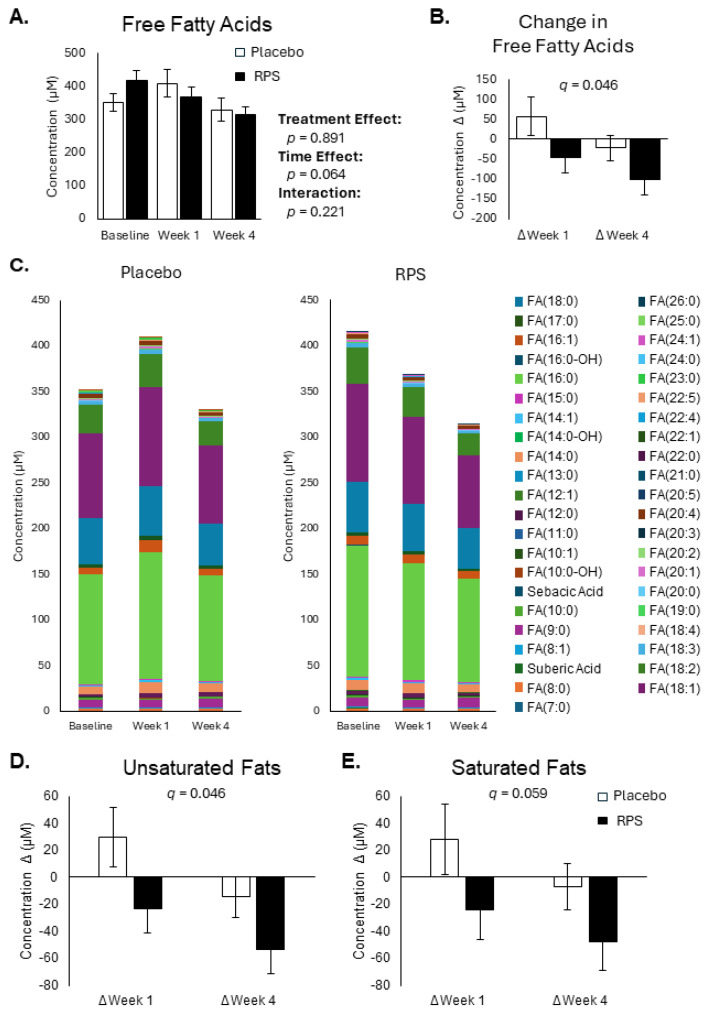
Effects of RPS and placebo on FFA levels. Total FFA levels were not different between placebo and RPS groups across baseline, week 1, and week 4 (**A**). RPS significantly reduced total FFA levels compared to placebo (**B**). Individual FFAs were similarly distributed in both placebo and RPS groups across baseline, week 1, and week 4 (**C**). Pooled unsaturated fats, including FA(8:1), FA(10:1), FA(12:1), FA(14:1), FA(16:1), FA(18:1), FA(18:2), FA(18:3), FA(18:4), FA(20:1), FA(20:2), FA(20:3), FA(20:4), FA(20:5), FA(22:1), FA(22:4), FA(22:5), FA(22:6), and FA(24:1), were reduced by RPS when compared to placebo (**D**). Saturated fats, including FA(7:0), FA(8:0), FA(9:0), FA(10:0), FA(11:0), FA(12:0), FA(13:0), FA(14:0), FA(15:0), FA(16:0), FA(17:0), FA(18:0), FA(19:0), FA(20:0), FA(21:0), FA(22:0), FA(23:0), FA(24:0), FA(25:0), and FA(26:0), were not significantly reduced in the RPS group compared to the placebo (**E**). See Table S1 for full analysis. (ANOVA; mean ± SEM).

While no differences were detected between treatment groups in total FFA levels at baseline, week 1, and week 4 (treatment effect *p* = 0.891; Figure 1A), a significant difference was observed between the changes in total FFA levels from the baseline, with RPS supplementation causing a general reduction in circulating FFA levels when compared to the placebo (*p* = 0.021, *q* = 0.046; Figure 1B). All reported FFAs were detected in both the placebo and RPS treatment groups, with the greatest contributions from hexadecanoic acid (FA16:0), octadecanoic acid (FA18:0), octadecaenoic acid (FA18:1), and octadecadienoic acid (FA18:2) (Figure 1C). RPS supplementation further led to a significant reduction in unsaturated FFAs (*p* = 0.016, *q* = 0.045; Figure 1D) but not saturated FFAs (*p* = 0.036, *q* = 0.059; Figure 1E) when compared to the placebo. Changes in several individual FFAs were also shown to be different between RPS and the placebo (Table S1). These included unsaturated FFAs octadecadienoic acid (FA(18:2); *p* = 0.008, *q* = 0.040), octadecatrienoic acid (FA(18:3); *p* = 0.002, *q* = 0.024), octadecatetraenoic acid (FA(18:4); *p* = 0.017, *q* = 0.046), eicosenoic acid (FA(20:1); *p* = 0.020, *q* = 0.046), eicosadienoic acid (FA(20:2) (*p* = 0.003, *q* = 0.025), eicosatrienoic acid (FA(20:3); *p* = 0.001, *q* = 0.024), eicosapentaenoic acid (FA(20:5); *p* = 0.009, *q* = 0.040), docosatetraenoic acid (FA(22:4); *p* = 0.013, *q* = 0.046), docosapentaenoic acid (FA(22:5); *p* = 0.005, *q* = 0.038), and docosahexaenoic acid (FA(22:6); *p* = 0.014, *q* = 0.46). Among the dicarboxylic FFAs, only concentrations of octanedioic acid (FA(8:0-DC); *p* = 0.025, *q* = 0.049) changed. Notably, the decreases were more pronounced at week 4 relative to week 1 for the RPS, suggesting a temporal reduction in circulating FFA levels with RPS.

### 3.2. Conjugated Secondary BAs Were Affected by RPS

**Figure 2:**
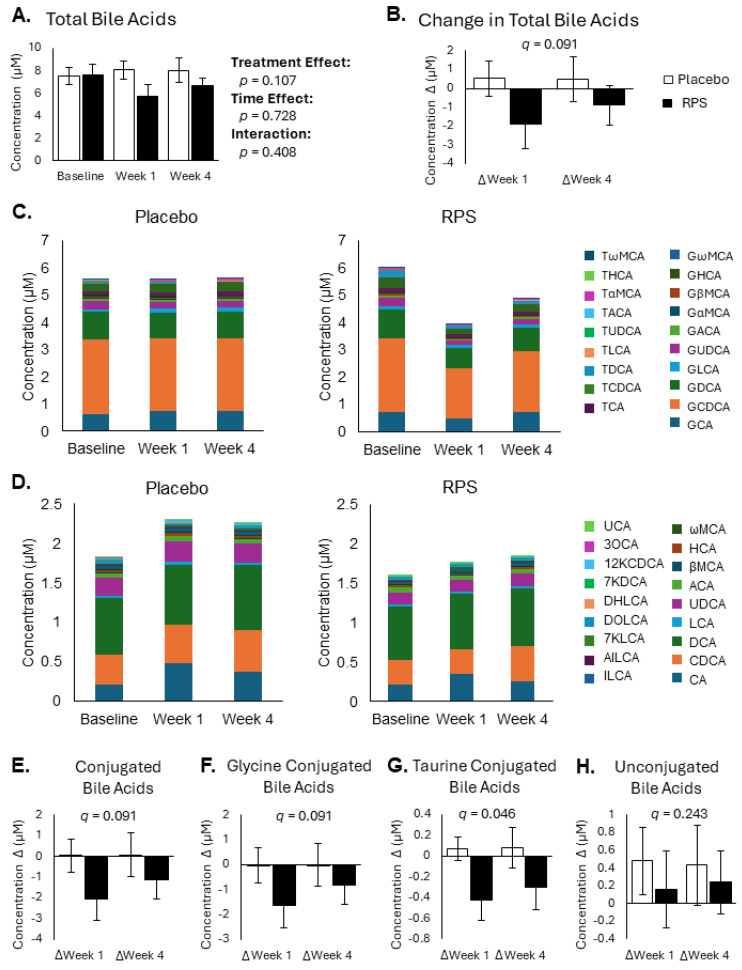
Effects of RPS and placebo on bile acid levels. Total bile acid levels were not significantly different between placebo and RPS groups across baseline, week 1 and week 4 (**A**). Changes in total bile acid levels tended to decrease in the RPS group compared to the placebo group, but this effect was not significant (**B**). Conjugated (**C**) and unconjugated (**D**) bile acid levels were similar in both treatment groups across baseline, week 1, and week 4. Total conjugated (**E**) and glycine-conjugated (**F**) bile acids tended to be reduced in the RPS group. RPS consumption significantly reduced taurine-conjugated bile acids (**G**) but had no effect on unconjugated bile acids (**H**). (ANOVA; mean ± SEM).

Bile acids regulate the digestion and absorption of dietary fats but are also heavily modified by gut microbiota and have been previously shown to influence host glucose and lipid metabolism in a structure-dependent manner [34,42,43,44]. There were no significant differences detected in total circulating BAs across baseline, week 1, and week 4 (treatment effect *p* = 0.107; Figure 2A). Changes in total BA levels were also unaffected by RPS (*p* = 0.091, *q* = 0.091; Figure 2B). All conjugated BAs (Figure 2C) and unconjugated BAs (Figure 2D), as measured by LC-MRM/MS, were detected in both groups. Among the total conjugated BAs (Figure 2E), glycine-conjugated BAs (Figure 2F), taurine-conjugated BAs (Figure 2G), and unconjugated BAs (Figure 2H), only taurine-conjugated BAs were reduced in the RPS group (*p* = 0.021, *q* = 0.046). Further analysis of individual BAs revealed that RPS specifically reduced serum levels of glycolithocholic acid (*p* = 0.022, *q* = 0.046), glycoallocholic acid (*p* = 0.021, *q* = 0.0046), taurodeoxycholic acid (*p* = 0.026, *q* = 0.049), taurolithocholic acid (*p* = 0.002, *q* = 0.024), and tauroallocholic acid (*p* = 0.008, *q* = 0.034) (Table S2).

### 3.3. Decreased FFA Levels Correlate with Reductions in Bile Acids

Given that both FFA and conjugated BA levels decreased after RPS supplementation, we concluded that as conjugated BAs tend to be more soluble relative to deconjugated BAs, conjugated BA reductions might explain FFA changes by altering lipid digestion. To test this hypothesis, we performed Pearson correlation analysis between week 4 changes in FFA levels and changes in conjugated BAs. No significant associations were observed between changes in total FFA levels and changes in conjugated, unconjugated, and total BAs for RPS or the placebo (Table S3), suggesting that FFA levels were not strongly impacted by global changes in the BA pool.

**Figure 3:**
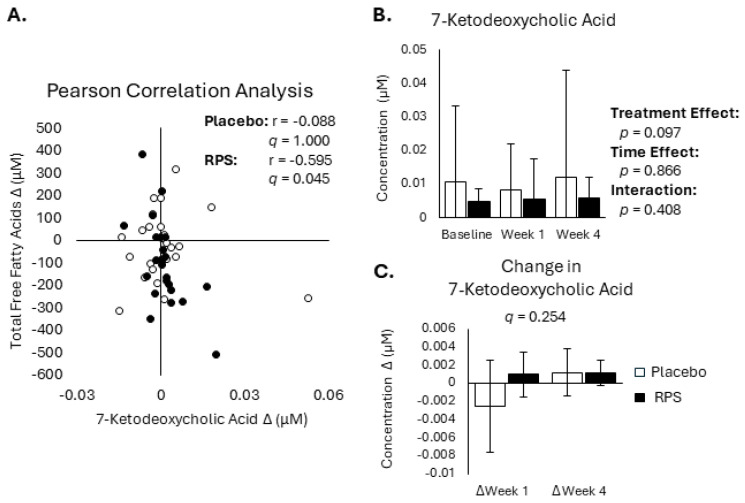
Effects of RPS and placebo on 7-ketodeoxycholic acid levels. Week 4 changes in 7-ketodeoxycholic acid are correlated with changes in FFA levels in the RPS but not placebo group (**A**). Levels of 7-ketodeoxycholic acid were not different between groups across baseline, week 1, or week 4 (**B**), nor were changes in 7-ketodeoxycholic acid between groups (**C**). (ANOVA; mean ± SEM).

Free fatty acid release from adipocytes is negatively regulated by insulin [5] and bile acids are known to modulate insulin-sensitive pathways [43], suggesting that RPS may decrease FFA levels by elevating one or more microbially modified secondary BA. We tested this reasoning by performing Pearson correlation analysis between changes in secondary BAs and changes in total FFA levels at week 4 for the placebo and RPS (Table S4). This analysis revealed that changes in 7-ketodeoxycholic acid were significantly correlated with changes in total FFAs for RPS (*r* = −0.530, *p* = 0.002, *q* = 0.045) but not the placebo (*r* = −0.088, *p* = 0.684, *q* = 1.00) (Figure 3A). However, 7-ketodeoxycholic acid was not different across groups at baseline, week 1, or week 4 (effect of treatment: *p* = 0.097; Figure 3B) and there was no difference in 7-ketodeoxycholic acid changes between groups (*p* = 0.594, *q* = 0.254; Figure 3C).

### 3.4. Changes in FFAs and Ketone Body Are Inter-Correlated

**Figure 4:**
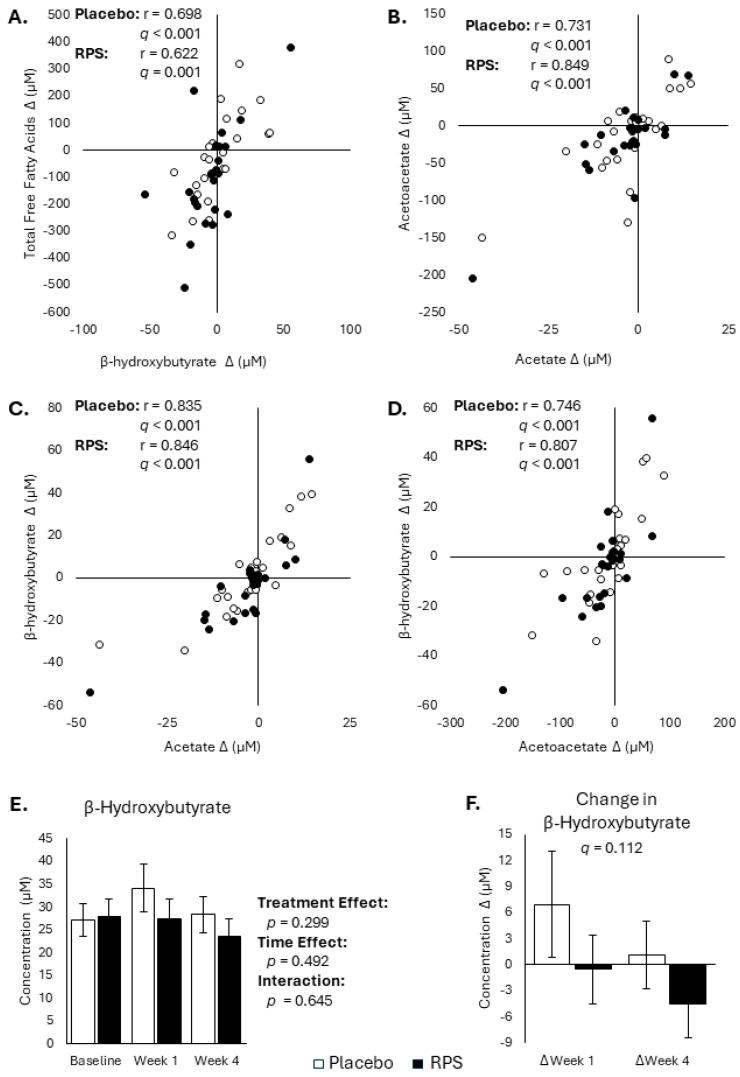
Effects of RPS and placebo on ketone bodies. Week 4 changes in β-hydroxybutyrate were significantly correlated with changes in FFA levels in both placebo and RPS (**A**) groups. Week 4 changes in acetoacetate were significantly correlated with changes in acetate levels in both placebo and RPS (**B**) groups. Week 4 changes in β-hydroxybutyrate were significantly correlated with changes in acetate levels in both placebo and RPS (**C**) groups. Week 4 changes in β-hydroxybutyrate were significantly correlated with changes in acetoacetate levels in both placebo and RPS (**D**) groups. β-hydroxybutyrate levels were not significantly different between placebo and RPS groups across baseline, week 1, or week 4 (**E**), nor were changes in β-hydroxybutyrate between treatment groups (**F**). (ANOVA; mean ± SEM).

Ketone bodies are metabolic intermediaries that serve as energy sources during times of starvation, but that can also act as signaling molecules to influence FFA metabolism [33]. Levels of acetate, acetoacetate, and β-hydroxybutyrate were comparable between the RPS and placebo treatment groups and ketone body levels were unaffected by RPS treatment (Table S5). However, week 4 changes in FFA levels were significantly correlated with changes in β-hydroxybutyrate for the placebo (*r* = 0.698; *p* < 0.001, *q* < 0.001) and RPS (*r* = 0.622; *p* = 0.001, *q* = 0.002) (Figure 4A). Shifts in ketone body levels were also shown to be highly inter-correlated, as was the case for acetoacetate and acetate, for both the placebo (*r* = 0.731, *p* < 0.001, *q* < 0.001) and RPS (*r* = 0.807, *p* < 0.001, *q* < 0.001) (Figure 4B–D). Despite being significantly correlated with changes in FFAs, β-hydroxybutyrate was not different across groups at baseline, week 1, and week 4 (Effect of treatment: *p* = 0.299; Figure 4E) and there was no difference in β-hydroxybutyrate changes between groups (*p* = 0.156, *q* = 0.112; Figure 4F).

### 3.5. Inter-Correlations between Changes in Select BAs, FFAs, and Ketone Bodies

**Figure 5:**
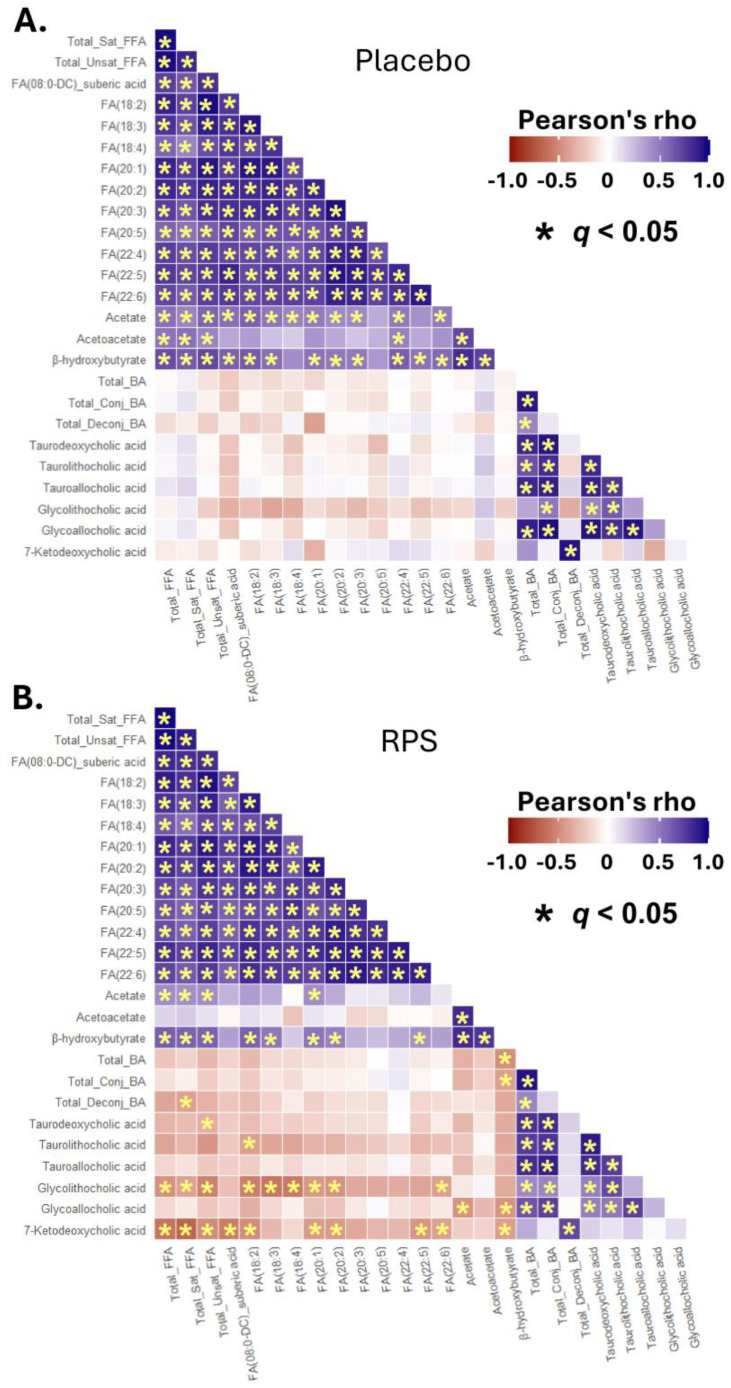
Interactions between FFAs, bile acids, and ketone bodies. Heatmap shows associations between week 4 changes in select FFAs, bile acids, and ketone bodies in the placebo (**A**) and RPS (**B**) groups. Color gradient indicates Pearson correlation coefficients; * *q* values < 0.05.

To gain a clearer understanding of the interactions between circulating levels of FFA, BAs, and ketone bodies, we systemically applied Peason correlations, including measures that either changed in response to RPS or associated with the RPS-induced reduction in total FFAs. This analysis revealed that week 4 changes in FFA levels were positively correlated with changes in ketone bodies but not changes in BAs in the placebo group (Figure 5A).

**Figure 6:**
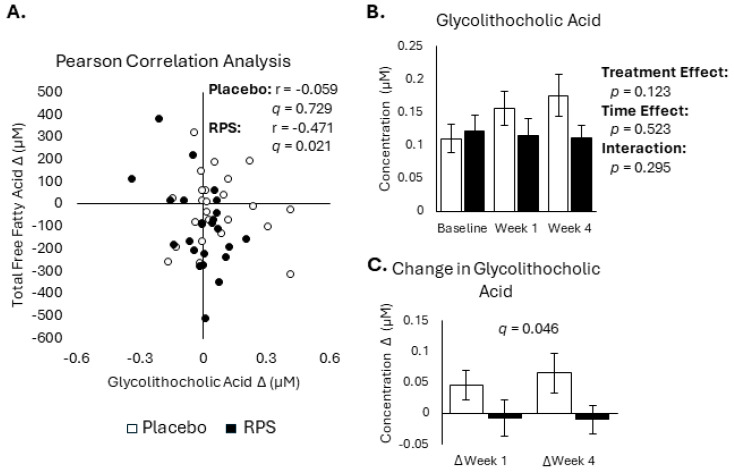
Effects of RPS and placebo on glycolithocholic acid levels. Week 4 changes in glycolithocholic acid are correlated with changes in FFA levels in the RPS group but not the placebo group (**A**). Levels of glycolithocholic acid were not significantly different between treatment group across baseline, week 1, and week 4 (**B**), but changes in glycolithocholic acid between treatment groups were significantly different (**C**). (ANOVA; mean ± SEM).

Similar association between FFA levels and ketone bodies were also observed for RPS; however, changes in several FFAs were shown to inversely correlate with week 4 changes in the BAs 7-ketodeoxycholic acid and glycolithocholic acid (Figure 5B and Figure 6A). Of note, further analysis of glycolithocholic acid showed no difference across groups at baseline, week 1, and week 4 (effect of treatment: *p* = 0.123; Figure 6B) but was shown to be elevated by the placebo (*p* = 0.022, *q* = 0.046; Figure 6C). Changes in 7-ketodeoxycholic acid and glycoallocholic acid were also inversely correlated with β-hydroxybutyrate for RPS but not the placebo (Figure 5). Overall, the number of significant correlations between FFAs and ketone bodies and FFA and BAs was shown to be different between treatment groups (Table S6). 

## 4. Discussion

Dietary supplementation with a low dose of RPS (3.5 g/day) led to significant reductions in total circulating levels of FFAs compared to the placebo group, which was driven mainly by reductions in unsaturated FFAs. RPS also reduced select conjugated secondary BAs, with taurine-conjugated BAs chiefly altered by supplementation. We further observed inverse correlations between RPS-induced reductions in total FFAs and changes in 7-ketodeoxycholic acid, even though 7-ketodeoxycholic acid levels were not different between RPS and the placebo. Changes in β-hydroxybutyrate were positively correlated with changes in FFAs in both groups, and levels of β-hydroxybutyrate tended to emulate the RPS-induced reductions in FFAs. Finally, correlation analysis revealed inverse relationships between FFAs and 7-ketodeoxycholic acid and glycolithocholic acid in the RPS group but not the placebo group. However, RPS supplementation is unlikely to stimulate improved insulin metabolism via elevated levels of these secondary BAs as their concentrations did not significantly change.

Previous clinical trials evaluating the effects of RS2 on metabolism have demonstrated improvements in insulin-dependent metabolic processes, albeit at high doses (40–80 g/day of RS-containing ingredients [19,27,45,46,47]). The minimum dose of RS required to achieve glycemic benefits was more recently estimated to be greater than 15 g/day [47]. The present study examined the effects of 2.1 g/day of RS2, provided as 3.5 g/day of RPS, which was an amount of RS2 substantially lower than that previously suggested for clinically relevant effects in humans. While RPS comprises native starch with a granular structure that is resistant to digestion and, thus, defined as a type 2 RS, its structure is unique when compared to other RS2s [10,35]. In contrast to high-amylose maize starch, RPSs tend to have lower proportions of amylose but with longer degrees of polymerization [48]. RPSs also tend to have higher levels of phosphorylated starch and larger diameter granules [49,50,51], with granule size previously shown to inversely associate with starch digestion [52]. However, further research is needed to determine whether the unique structural attributes of RPS contributed to the observed reductions in circulating FFA levels.

Supplementation of 3.5 g of RPS per day was previously reported to increase *Akkermansia* levels [17], as do other doses and forms of RS [13,20,53]. Given that *Akkermansia* plays a role in stimulating GLP-1 secretion [54], and that RPS was previously shown in a Western diet T2D swine model to elevate GLP-1 levels, decrease fasting blood glucose, and attenuate insulin resistance [55], we may speculate that reduced FFA levels are a consequence of enhanced insulin signaling due to elevated GLP-1, which might be driven by an enrichment of *Akkermansia* in those consuming RPS. However, future trials examining the effect of RPS on GLP-1, insulin sensitivity, and fasting blood glucose in people with insulin resistance are warranted to confirm these effects.

Given that macronutrient intake did not change over time in either treatment group as previously described [32], it is unlikely that decreases in FFAs are attributed to habitual dietary changes. Additionally, most dietary FFAs are absorbed by enterocytes, and repackaged as phospholipids or cholesterol esters for transport in the blood [56], further suggesting that any impact of diet on FFA levels would be minimal. Resistant potato starch-dependent decreases in FFAs are also unlikely to be due to reduced fat absorption resulting from reductions in more hydrophobic conjugated BAs because changes in conjugated BAs were not correlated with changes in total FFA levels.

Previous studies have demonstrated the role of microbially modified secondary BAs as signaling molecules that influence host metabolism, including insulin signaling and glucose metabolism [43]. Thus, changes in BA pool configurations induced during intestinal fermentation of prebiotics such as RPS could induce systemic metabolic effects [57]. Changes in 7-ketodeoxycholic acid and glycolithocholic acid were inversely correlated with changes in FFAs in individuals consuming RPS, suggesting that shifts in the abundance of these BAs might be influencing or be influenced by the decreased FFA levels. While there were no significant differences between treatment groups for 7-ketodeoxycholic acid, glycolithocholic acid was significantly increased in the placebo group, making it unlikely that these BAs are acting as metabolically beneficial signaling molecules during RPS supplementation. FFAs and BAs were only correlated in the RPS treatment group, suggesting that FFA decreases are related to RPS-dependent changes in the BA pool. While ketone bodies were correlated with FFAs in both treatment groups, there were fewer correlations in the RPS group, suggesting that RPS is somehow disrupting these interactions. Overall, further research utilizing preclinical models is required to determine the nature of these mechanistic relationships and their relevancy to host metabolism.

## 5. Conclusions

Low-dose RPS supplementation beneficially influenced measures of fatty acid metabolism in a manner consistent with previous studies using high doses of RS [13,30]. This effect was correlated to changes in microbially modified secondary BAs, suggesting that RPS may achieve this effect at least in part by influencing enterohepatic circulation and the activity of microbial enzymes responsible for shaping the BA pool. Dietary supplement and functional food innovation requires ingredient providers to substantiate clinical benefits at dosages that are compatible with commercial formats and prices so that these benefits can ultimately be realized by consumers. Therefore, high RS doses that demonstrate metabolic improvements represent a challenge from a product development and commercialization standpoint. The findings from this study are relevant as they present evidence suggesting that 3.5 g of RPS per day can improve FFA levels, an early marker of insulin resistance. Future clinical trials evaluating the efficacy of formulation-friendly low doses of RPS in patients with prediabetes to prevent progression to T2D are warranted.

## 6. Patents

The sister company of MSP Starch Products Inc., McPharma Biotech Inc., holds relevant patents US11058711B2, CA3024201A1, AU2017294806A1, and provisional patent applications PCT/CA2023/050884 and 63/622,713.

## Data Availability

The data presented in this study are available on request from the corresponding author, but the data are owned by MSP Starch Products Inc. and restrictions apply to the use of these data, including the execution of nondisclosure agreements and/or material transfer agreements.

## Supplementary Materials

The following supporting information can be downloaded at https://www.mdpi.com/article/10.3390/metabo14100536/s1: Tables S1–S6. Table S1. Mean changes in individual FFA levels in RPS- and placebo-consuming individuals at week 1 and week 4 (Two-way ANOVA). Table S2. Mean changes in bile acid levels in RPS- and placebo-consuming individuals at week 1 and week 4 (Two-way ANOVA). Table S3. Pearson correlation analysis comparing changes in total FFA levels and changes in different groups of bile acids at week 4. Table S4. Pearson correlation analysis comparing changes in FFA levels with changes in microbially-modified bile acids at week 4. Table S5. Mean changes in ketone body levels in RPS- and placebo-consuming individuals at week 1 and week 4 (Two-way ANOVA). Table S6. Differences in the number of significant correlations between FFAs, BAs, and ketone bodies between treatment groups.
