# Supplementary material for: Resistant Potato Starch Supplementation Reduces Serum Free Fatty Acid Levels and Influences Bile Acid Metabolism
Source: Metabolites. 2024 Oct 5;14(10):536. doi: 10.3390/metabo14100536 (PMC11510092; doi:10.3390/metabo14100536)
Supplement: Supplementary file 1 [file metabolites-14-00536-s001.zip › metabolites-3209571-supplementary.pdf]

Table S1. Mean changes in individual FFA levels in RPS- and placebo-consuming individuals at week 1 and week 4 (Two-way ANOVA).

Lipid Number	Systematic Name	Week 1 Change (µM)		Week 4 Change (µM)		p value	q value
		Placebo	RPS	Placebo	RPS		
FA(7:0)	Heptanoic Acid	-0.03252	-0.0734	-0.02306	-0.01506	0.548	0.243
FA(8:0)	Octanoic Acid	-0.20955	-0.79991	0.25651	-0.46205	0.268	0.158
FA(8:0-DC)	Octanedioic Acid	0.16302	-0.09211	0.08872	-0.06627	0.025	0.049
FA(8:1)	Octadecenoic Acid	-0.15009	-0.24575	-0.03869	-0.21164	0.111	0.100
FA(9:0)	Nonanoic Acid	-0.66658	-2.02864	0.48457	0.02959	0.196	0.126
FA(10:0)	Decanoic Acid	0.02549	-0.43559	0.10326	-0.47096	0.279	0.162
FA(10:0-DC)	Decanedioic Acid	0.00210	0.00104	0.00082	-0.00005	0.438	0.222
FA(10:0-OH)	Hydroxydecanoic Acid	0.01918	-0.00212	-0.01133	0.00126	0.800	0.327
FA(10:1)	Decaenoic Acid	0.12784	0.0518	0.01348	-0.11908	0.139	0.109
FA(11:0)	Undecanoic Acid	-0.00021	-0.02305	-0.00144	-0.01661	0.133	0.108
FA(12:0)	Dodecanoic Acid	1.82406	0.73445	2.18205	-1.08397	0.119	0.105
FA(12:1)	Dodecaenoic Acid	0.05506	0.00360	0.01302	-0.0178	0.089	0.091
FA(13:0)	Tridecanoic Acid	0.02713	0.00274	0.00928	-0.00691	0.233	0.144
FA(14:0)	Tetradecanoic Acid	3.22342	-0.59344	0.8653	-2.45013	0.039	0.059
FA(14:0-OH)	Hydroxytetradecanoic Acid	0.00280	-0.00092	-0.00099	-0.00276	0.061	0.075
FA(14:1)	Tetradecaenoic Acid	1.07067	-0.08563	0.03441	-0.36912	0.094	0.091
FA(15:0)	Pentadecanoic Acid	0.40782	-0.04902	0.04199	-0.24299	0.099	0.094
FA(16:0)	Hexadecanoic Acid	18.1881	-16.1377	-5.23673	-30.7034	0.041	0.059
FA(16:0-OH)	Hydroxyhexadecanoic Acid	0.14986	0.03241	-0.06489	-0.10468	0.200	0.126
FA(16:1)	Hexadecaenoic Acid	5.69467	-0.64908	0.22127	-2.33232	0.068	0.081
FA(17:0)	Heptadecanoic Acid	0.90317	-0.04301	-0.45295	-1.19077	0.135	0.108
FA(18:0)	Octadecanoic Acid	4.80388	-4.1413	-3.94963	-10.724	0.142	0.109
FA(18:1)	Octadecaenoic Acid	15.4498	-12.5437	-7.4382	-27.9825	0.028	0.050
FA(18:2)	Octadecadienoic Acid	4.13813	-7.02025	-5.51724	-15.9776	0.008	0.040
FA(18:3)	Octadecatrienoic Acid	1.57358	-1.11114	-0.37364	-2.49709	0.002	0.024
FA(18:4)	Octadecatetraenoic Acid	0.03058	-0.01574	-0.01293	-0.03785	0.017	0.046
FA(19:0)	Nonadecanoic Acid	0.016116	-0.01162	-0.01845	-0.065	0.058	0.075
FA(20:0)	Eicosanoic Acid	-0.1266	-0.09391	-0.25131	-0.13225	0.453	0.224
FA(20:1)	Eicosenoic Acid	0.47864	-0.4136	-0.3772	-0.86386	0.020	0.046
FA(20:2)	Eicosadienoic Acid	0.27076	-0.12517	-0.05707	-0.3976	0.003	0.025
FA(20:3)	Eicosatrienoic Acid	0.17027	-0.11532	-0.05513	-0.2626	0.001	0.024
FA(20:4)	Eicosatetranoic Acid	0.08110	-0.49358	-0.77743	-1.07034	0.055	0.075
FA(20:5)	Eicosapentanoic Acid	0.05703	-0.04668	-0.02901	-0.0803	0.009	0.040
FA(21:0)	Heneicosanoic Acid	-0.00242	-0.00366	-0.00413	-0.00495	0.742	0.314
FA(22:0)	Docosanoic Acid	-0.34739	-0.1852	-0.47626	-0.04836	0.145	0.109
FA(22:1)	Docosaenoic Acid	0.00517	-0.01405	-0.03328	-0.03184	0.541	0.243
FA(22:4)	Docosatetraenoic Acid	0.03194	-0.01061	-0.02101	-0.05504	0.013	0.046
FA(22:5)	Docosapentaenoic Acid	0.23549	-0.0794	-0.04656	-0.24303	0.005	0.038
FA(22:6)	Docosahexaenoic Acid	0.42487	-0.2142	-0.30047	-0.62026	0.014	0.046
FA(23:0)	Tricosanoic Acid	-0.0084	-0.00369	-0.014	-0.00797	0.538	0.243
FA(24:0)	Tetracosanoic Acid	-0.26138	-0.14821	-0.44055	-0.0466	0.176	0.119
FA(24:1)	Tetracosaenoic Acid	0.02070	0.00716	-0.005	-0.01312	0.447	0.223
FA(25:0)	Pentacosanoic Acid	-0.01305	0.002757	-0.01179	-0.01246	0.431	0.222
FA(26:0)	Hexacosanoic Acid	-0.05373	-0.00692	-0.06906	-0.01168	0.091	0.091

Table S2. Mean changes in bile acid levels in RPS- and placebo-consuming individuals at week 1 and week 4 (Two-way ANOVA).

Bile Acid	Week 1 Change (µM)		Week 4 Change (µM)		p value	q value
	Placebo	RPS	Placebo	RPS		
Cholic Acid	0.27219	0.13421	0.16117	0.04649	0.508	0.238
Chenodeoxycholic Acid	0.11366	0.00567	0.15580	0.12407	0.593	0.254
Deoxycholic Acid	0.02934	0.01596	0.09504	0.05771	0.830	0.336
Lithocholic Acid	0.00570	0.00440	0.00377	0.00466	0.958	0.375
Ursodeoxycholic Acid	0.03539	-0.00075	0.01468	0.01405	0.490	0.238
Allocholic Acid	0.00627	-0.00401	0.00227	-0.01322	0.270	0.160
β-muricholic Acid	0.00026	-0.00045	0.00041	-0.00024	0.378	0.213
Hyocholic Acid	0.00890	0.00137	0.00006	0.00070	0.425	0.222
ω-muricholic Acid	-0.00064	-0.00064	-0.00345	0.00094	0.554	0.243
Isolithocholic Acid	0.00549	0.00277	0.00396	0.00103	0.936	0.372
Alloisolithocholic Acid	0.00122	0.00200	-0.00166	0.00388	0.184	0.123
7-Ketolithocholic Acid	0.00061	-0.00334	0.00526	-0.00348	0.417	0.222
Dioxolithocholic Acid	0.00580	0.00152	0.00115	0.00205	0.434	0.222
Dehydrolithocholic Acid	0.00128	0.0012	0.00055	0.00106	0.785	0.327
7-Ketodeoxycholic Acid	-0.00254	0.00097	0.00118	0.00112	0.594	0.254
12-Ketochenodeoxycholic Acid	0.00020	-0.00168	-0.00327	-0.0032	0.797	0.327
3-Oxocholic Acid	0.00010	0.00028	0.00028	0.00001	0.939	0.372
Ursocholic Acid	-0.00277	0.00058	-0.00325	0.00057	0.154	0.112
Glycocholic Acid	0.08613	-0.23564	0.11743	0.02746	0.168	0.118
Glycochenodeoxycholic Acid	-0.07691	-0.8668	-0.08732	-0.50755	0.175	0.119
Glycodeoxycholic Acid	-0.04148	-0.32085	-0.02612	-0.19683	0.271	0.160
Glycolithocholic Acid	0.04913	-0.00756	0.06511	-0.01023	0.022	0.046
Glycoursodeoxycholic Acid	-0.06081	-0.13551	-0.07458	-0.09459	0.418	0.222
Glycoallocholic Acid	0.00571	-0.03739	0.00154	-0.01909	0.021	0.046
Glyco-α-muricholic Acid	-0.00064	-0.00346	-0.00203	-0.00202	0.409	0.222
Glyco-β-muricholic Acid	0.00615	-0.03141	-0.00587	-0.02723	0.070	0.081
Glycohyocholic Acid	-0.00009	-0.01553	-0.00921	-0.00507	0.502	0.238
Glyco-ω-muricholic Acid	0.00026	-0.00371	-0.00153	-0.00291	0.109	0.100
Taurocholic Acid	0.01245	-0.05264	0.03701	-0.04735	0.040	0.060
Taurochenodeoxycholic Acid	0.02242	-0.16796	0.03092	-0.08607	0.032	0.056
Taurodeoxycholic Acid	0.01879	-0.13229	0.01974	-0.10496	0.026	0.049
Taurolithocholic Acid	0.00460	-0.00785	0.00626	-0.00691	0.002	0.024
Tauroursodeoxycholic Acid	-0.00085	-0.0148	-0.00409	-0.01057	0.060	0.075
Tauroallocholic Acid	0.00128	-0.00464	0.00068	-0.00309	0.008	0.040
Tauro- α-muricholic Acid	0.00954	-0.03547	-0.00339	-0.0349	0.084	0.091
Taurohyocholic Acid	0.00156	-0.0045	-0.00081	-0.00288	0.133	0.108
Tauro- ω-muricholic Acid	0.00020	-0.00344	-0.00212	-0.00286	0.195	0.126

Table S3. Pearson correlation analysis comparing changes in total FFA levels and changes in different groups of bile acids at week 4.

Group	Placebo			RPS		
	Pearson (r)	p value	q value	Pearson (r)	p value	q value
All Conjugated Bile Acids	0.033	0.878	0.729	-0.134	0.533	0.280
All Deconjugated Bile Acids	-0.144	0.501	0.593	-0.375	0.071	0.062
All Bile Acids	-0.026	0.905	0.734	-0.245	0.249	0.166

Table S4. Pearson correlation analysis comparing changes in FFA levels with changes in microbially-modified bile acids at week 4.

Bile Acid	Placebo			RPS		
	Pearson (r)	p value	q value	Pearson (r)	p value	q value
CA	-0.278	0.188	1.000	-0.270	0.201	0.286
CDCA	-0.258	0.223	1.000	-0.270	0.201	0.286
DCA	0.269	0.204	1.000	-0.437	0.033	0.180
LCA	0.433	0.034	0.983	-0.428	0.037	0.180
UDCA	-0.161	0.453	1.000	-0.040	0.854	0.605
ACA	0.109	0.613	1.000	0.005	0.983	0.639
βMCA	0.090	0.675	1.000	0.031	0.884	0.605
HCA	0.052	0.811	1.000	-0.388	0.061	0.203
ωMCA	0.048	0.824	1.000	-0.325	0.121	0.238
TDCA	0.047	0.826	1.000	-0.314	0.136	0.256
TLCA	0.031	0.884	1.000	-0.380	0.067	0.208
TUDCA	0.055	0.799	1.000	0.058	0.787	0.582
TACA	0.095	0.660	1.000	-0.174	0.417	0.430
TαMCA	0.170	0.426	1.000	-0.291	0.168	0.275
THCA	0.147	0.493	1.000	-0.233	0.272	0.335
TωMCA	0.120	0.576	1.000	-0.234	0.272	0.335
GDCA	0.070	0.746	1.000	-0.310	0.141	0.261
GLCA	-0.059	0.784	1.000	-0.471	0.020	0.180
GUDCA	-0.022	0.920	1.000	0.033	0.878	0.605
GACA	0.037	0.864	1.000	-0.166	0.440	0.435
GαMCA	0.139	0.516	1.000	-0.370	0.075	0.208
GβMCA	0.031	0.886	1.000	-0.379	0.068	0.208
GHCA	0.181	0.396	1.000	-0.075	0.728	0.558
GωMCA	0.151	0.481	1.000	-0.351	0.093	0.208
12KCDCA	-0.034	0.873	1.000	0.011	0.959	0.631
DOLCA	0.017	0.938	1.000	0.214	0.315	0.367
7KDCA	-0.088	0.684	1.000	-0.595	0.002	0.045
7KLCA	-0.246	0.0247	1.000	-0.387	0.062	0.203
3OCA	-0.154	0.473	1.000	-0.272	0.198	0.286
DHLCA	0.320	0.128	1.000	-0.468	0.021	0.180
ILCA	-0.157	0.464	1.000	-0.268	0.206	0.286
UCA	0.343	0.101	1.000	-0.336	0.108	0.235
AILCA	-0.235	0.268	1.000	0.020	0.926	0.626

Table S5. Mean changes in ketone body levels in RPS- and placebo-consuming individuals at week 1 and week 4 (Two-way ANOVA).

Ketone Body	Week 1 Change (µM)		Week 4 Change (µM)		p value	q value
	Placebo	RPS	Placebo	RPS		
Acetate	-0.4086	-3.3442	-2.9693	-3.6199	0.502	0.238
Acetoacetate	28.4223	8.2212	-12.1791	-19.1582	0.300	0.171
β-Hydroxybutyrate	6.9249	-0.5336	1.1457	-4.5100	0.156	0.112

Table S6. Differences in the number of significant correlations between FFAs, BAs, and ketone bodies between treatment groups.

Comparison	Placebo		RPS		p value
	Significant	Not significant	Significant	Not significant	
FFA vs Ketone Body	28	14	12	30	0.001
FFA vs BA	0	135	21	114	<0.001
Ketone Body vs BA	0	27	5	22	0.051
